# A Toolbox for Tuberculosis Diagnosis: An Indian Multicentric Study (2006-2008): Microbiological Results

**DOI:** 10.1371/journal.pone.0043739

**Published:** 2012-08-24

**Authors:** Philippe H. Lagrange, Satheesh K. Thangaraj, Rajeshwar Dayal, Alka Despande, Nirmal K. Ganguly, Enrico Girardi, Beenu Joshi, Kiran Katoch, Vishwa M. Katoch, Manoj Kumar, Vemu Lakshmi, Marc Leportier, Christophe Longuet, Subbalaxmi V. S. Malladi, Deepali Mukerjee, Deepthi Nair, Alamelu Raja, Balambal Raman, Camilla Rodrigues, Pratibha Sharma, Amit Singh, Sarman Singh, Archana Sodha, Basirudeen Syed Ahamed Kabeer, Guy Vernet, Delia Goletti

**Affiliations:** 1 Service de Microbiologie, Hôpital Saint Louis, Assistance Publique- Hôpitaux de Paris, Paris VII Denis Diderot University, Paris, France; 2 bioMérieux, Marcy-l’Etoile, France, and New Delhi, India; 3 SN Medical College, Agra, India; 4 JJ Hospital, Grant Medical College, Mumbai, India; 5 Indian Council of Medical Research (ICMR), New Delhi, India; 6 Department of Epidemiology and Preclinical Research, L. Spallanzani National Institute for Infectious Diseases (INMI), Rome, Italy; 7 National JALMA Institute of Leprosy & Other Mycrobacterial Diseases (ICMR), Agra, India; 8 All India Institute of Medical Sciences, New Delhi, India; 9 Nizam’s Institute of Medical Sciences, Hyderabad, India; 10 Fondation Mérieux, Lyon, France; 11 Safdarjung Hospital, New Delhi, India; 12 Tuberculosis Research Centre (TRC), ICMR, Cheput, Chennai, India; 13 P.D. Hinduja National Hospital and Research Center, Mumbai, India; Johns Hopkins University School of Medicine, United States of America

## Abstract

**Background:**

The aim of this multicentric prospective study in India was to assess the value of several microbiological tools that contribute to the diagnosis of tuberculosis (TB) according to HIV status.

**Methods:**

Standard microbiological tools on individual specimens were analyzed.

**Results:**

Among the 807 patients with active TB, 131 were HIV-infected, 316 HIV-uninfected and 360 had HIV-unknown status. Among the 980 non-active TB subjects, 559 were at low risk and 421 were at high risk of *M. tuberculosis* (Mtb) exposure. Sensitivity of smear microscopy (SM) was significantly lower in HIV-infected (42.2%) than HIV-uninfected (75.9%) (p = 0.0001) and HIV-unknown pulmonary TB patients (61.4%) (p = 0.004). Specificity was 94.5% in non-TB patients and 100% in health care workers (HCW) and healthy family contacts. Automated liquid culture has significantly higher diagnostic performances than solid culture, measured by sensitivity (74.7% *vs.* 55.9%) (p = 0.0001) and shorter median time to detection (TTD) (12.0 vs. 34.0 days) (p = 0.0001). Specificity was 100% in HCW and cured-TB patients, but was lower in non-TB patients (89%) due to isolation of Mycobacteria other than tuberculosis (MOTT). TTD by both methods was related to AFB score. Contamination rate was low (1.4%). AccuProbe hybridization technique detected *Mtb* in almost all culture-positive specimens, but MOTT were found in 4.7% with a significantly higher frequency in HIV-infected (15%) than HIV-uninfected TB patients (0.5%) (p = 0.0007). Pre-test classification significantly increased the diagnostic value of all microbiological tests in pulmonary TB patients (p<0.0001) but to a lesser degree in extrapulmonary TB patients.

**Conclusions:**

Conventional microbiological tools led to results similar to those already described in India special features for HIV-infected TB patients included lower detection by SM and culture. New microbiological assays, such as the automated liquid culture system, showed increased accuracy and speed of detection.

## Introduction

Tuberculosis (TB) is a major global health problem. In 2010, 8.8 million incident cases of TB and 1.1 million deaths were reported by the World Health Organization (WHO) [1]. In India 2.3 million new TB cases are estimated to have occurred in 2010, accounting for one-quarter of the world’s new TB cases [1]. This makes India the country with the highest TB burden in the world. It has been estimated that in 2010, only 41% of the 1,522,147 million notified were smear positive, and that two out of five Indians were infected with *M.tuberculosis* [2]. There are an estimated 2.5 million Indians living with HIV infection. Regarding smear-positive TB, HIV infection was 1.4 times more likely to occur among smear-negative patients and 1.3 times more likely among extrapulmonary patients [3].

**Figure 1:**
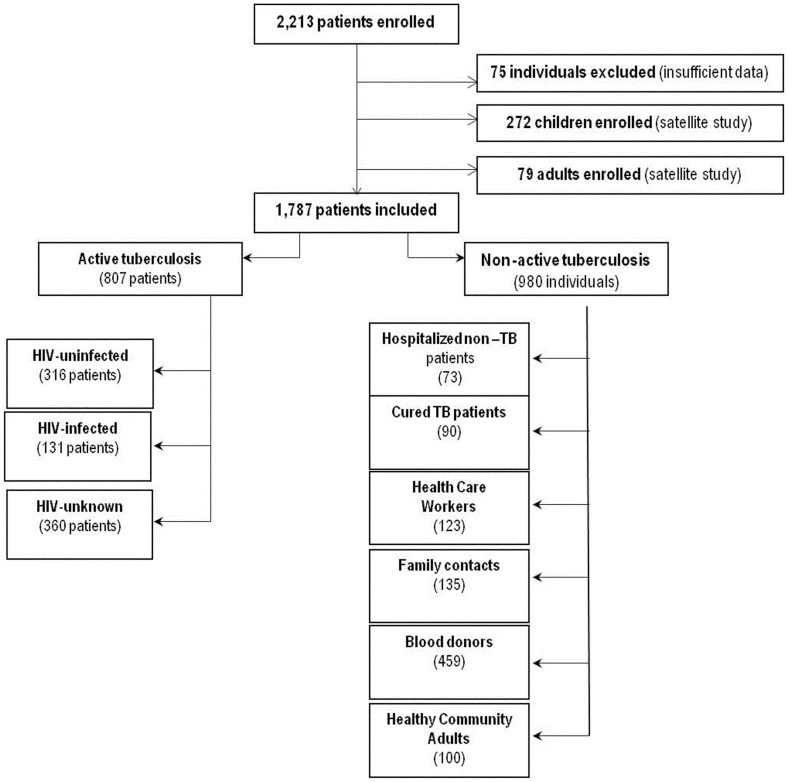
Flow chart of patients recruited to the multicentric study stratified by patient subgroups.

The basis of TB control programs consists of timely diagnosis and correct treatment of patients with active TB: early diagnosis is essential for controlling the spread of this disease.

Diagnosis of TB relies mostly on the detection of acid-fast bacilli by microscopy (smear) and less frequently by culture. Microscopy is rapid, usually specific, and inexpensive but has low sensitivity [4]–[5] that may be improved using new sputum processing and fluorescence microscopy [6]. Culture is more sensitive, but it can take several weeks to obtain results. In addition, solid culture may be falsely negative in 10–30% of cases [7]. Faster and more accurate diagnostic tests are needed to better control TB [8]. Nucleic acid amplification tests, which can give results in a few hours, have been developed to address these issues [9]–[11].

The development of real-time PCR testing platforms has significantly improved PCR technologies, the advantages of which are shortened turnaround time, automation of the procedure that reduces hands-on time and decreased risk of cross-contamination [12]. Recently, Cepheid, (Sunnyvale, CA, USA) developed a real-time PCR test (Xpert MTB/RIF) on the GeneXpert System that simultaneously detects both MTB and Rifampin resistance [13]. Recent studies reported high sensitivity and specificity of the test in respiratory specimens collected from patients living in countries with high and low prevalence of TB [14], [15].

Therefore the aim of the first part of the multicentric prospective study in India was to assess the value of several microbiological tools that contribute to TB diagnosis according to HIV-status. The second part will involve the immunological assays (manuscript in preparation).

**Table 1:** Demographic and clinical characteristics of the patients with active tuberculosis.

	Patients with active tuberculosis (n = 807)					
	Pulmonary tuberculosis (n = 656)			Extrapulmonary tuberculosis (n = 151)		
	HIV-infected	HIV-uninfected	HIV-unknown	HIV-infected	HIV-uninfected	HIV-unknown
	(n = 93)(n = 270)(n = 293)(n = 38)(n = 46)(n = 67)					
	Percentage					
**Disease Localization**	71.0	85.4	81.4	29.0	14.6	18.6
**Median Age (IQR)**	35.0	34.0	30.0	36.0	30.0	28.0
**Ratio M/F**	4.8	2.3	2.3	5.3	1.7	1.0
**Male prevalence**	82.8	66.3	70.0	84.2	63.0	50.8
**Diabetes**	0.0	11.1	2.4	0.0	6.5	1.5
**Poor Nutrition**	0.0	1.1	10.9	0.0	0.0	4.5
**Past TB**	28.4^(a)^	15.2	21.4	26.5^(c)^	10.9	5.9
TB <2 years	13.4	6.0	9.2	11.8	4.4	4.4
TB 2 to10 years	14.9	7.1	9.2	14.7	4.4	1.5
TB>10 years	0.0	2.2	3.1	0.0	2.2	0.0
**Contact with TB**	12.9^(b)^	8.5	8.2	12.5^(d)^	19.6	6.0

a 26/93 patients have no record;

b 23/93 patients have no record;

c 4/38 patients have no record;

d 6/38 patients have no record.

**Table 2:** Extrapulmonary TB localizations according to HIV status.

	HIV-infected	HIV-uninfected	HIV-unknown	Total
	(n = 38)(n = 46)(n = 67)(n = 151)			
	Percentage (95% CI)			
**Cervical lymphadenopathy**	5.3 (1.4−17.6)	30.4 (18.9–45.1)	32.8 (22.6–45.0)	25.2 (18.8–32.8)
**Mediastinal lymphadenopathy**	7.9 (2.7−21;1)	0.0 (0.0–8.0)	0.0 (0.0–5.6)	2.0 (0.7–5.8)
**Pleural**	27.8 (13.7−44.3)	26.1 (15.4–40.6)	19.4 (11.6–30.7)	23.2 (17.1–30.7)
**Abdominal**	29.0 (16.8−45.1)	17.4 (9.0–31.0)	7.5 (3.2–16.5)	15.9 (10.8–22.7)
**CNS**	15.8 (7.3−30.8)	15.2 (7.5–28.5)	0.0 (0.0–5.6)	8.6 (5.1–14.3)
**Disseminated**	10.5 (4.1−27.5)	2.2 (0.4–11.6)	0.0 (0.0–5.6)	3.3 (1.4–7.6)
**Skeletal**	5.3 (1.4−17.6)	2.2 (0.4–11.6)	38.8 (27.9–51.0)	19.2 (13.6–26.4)
**Genito**–**urinary**	0.0 (0.0–9.5)	2.2 (0.4–11.6)	0.0 (0.0–5.6)	0.7 (0.1–3.8)
**Skin**	0.0 (0.0–9.5)	0.0 (0.0–8.0)	2.2 (0.4–11.6)	0.7 (0.1–3.8)
**Rectal**	0.0 (0.0–9.5)	2.2 (0.4–11.6)	0.0 (0.0–5.6)	0.7 (0.1–3.8)
**Pericarditis**	0.0 (0.0–9.5)	2.2 (0.4–11.6)	0.0 (0.0–5.6)	0.7 (0.1–3.8)

TB: tuberculosis, CNS: central nervous system; TB: HIV: Human Immunodeficiency Virus; CI: confidence interval.

## Materials and Methods

### Study Populations

Adults and children with suspected active TB were prospectively enrolled from January 2006 to July 2008 with a real capability of a six month follow-up with antituberculous therapy, as a strict inclusion criterion. Nine centers were involved: four tertiary care centers: 3 public (AIIMS and Safdarjung Hospital, New Delhi; Nizam’s Institute of Medical Sciences, Hyderabad) and 1 private (P D Hinduja Hospital and medical Research Centre, Mumbai), two referral centers (JALMA, Agra; Tuberculosis Research Centre-TRC, Chennai), one pediatric hospital (SN Medical College, Agra) and two centers caring for HIV-infected patients (JJ hospital, Mumbai; Government Hospital for Thoracic Medicine, Chennai).

Non-active TB controls were also enrolled: blood donors from three centers (AIIMS, Hinduja and Nizam’s), healthy community adults (HCA-check up individuals) from Hinduja outpatients, hospitalized non-active-TB patients from three centers (Hinduja, AIIMS and Safdarjung), leprosy patients from one center (JALMA), healthy family contacts (HFC) from 2 centers (JALMA, TRC), health care workers (HCW-laboratory staff and nurses) from three centers (Hinduja, AIIMS and TRC), and cured TB patients from one center (TRC).

All enrolled individuals were stratified by risk for TB (defined as previous exposure to active TB cases, past TB, diabetes, poor nutritional conditions, immunological or other debilitating conditions) and therefore non-active TB controls were divided into “low TB risk" (blood donors and healthy community adults) and “high TB risk" (HCW, healthy family contacts, cured TB and no TB patients). Subjects at risk of TB were included with one condition: a real capability of a six month follow-up to exclude occurrence of active TB. The HIV-infected subjects were not undergoing antiretroviral therapy.

### Clinical and Chest X-Ray Examinations

Clinical symptoms and radiological findings were first assessed independently by each clinician taking part in the enrollment. Each individual’s data were initially recorded at each site using a standardized questionnaire involving 3 files (clinical/radiological evaluation, clinical/radiological follow-up and laboratory analysis) for patients with active TB and control groups (except for blood donors). The clinical TB suspicion (CSTB) score was done by the clinician in charge of the patient (3 categories were selected: very high, high, low), as previously reported [16].

Written informed consent was obtained from all patients before enrolment. Parents signed the written consent for the children population. The study was approved by the local ethical committees: the Institutional Ethical Committee of Tuberculosis Research Centre in Chennai (TRC-IEC No: 2006005), the Institutional Review Board at Hinduja Hospital, Mumbai (No: 316-05-CR), the Institutional Ethical Committee of AIIMS, New Delhi (AIIMS-IEC No: A-35∶05/10/2005), the Institutional Ethical Committee of the Safdarjung Hospital, New Delhi (N° Sur./1/2007), the NIMS Institutional Ethics Committee (No.EC/264 (A) /2005) and the Ethical Committee of National JALMA Institute for Leprosy and others Mycobacterial Diseases, Agra (minutes: 27/04/2006).

**Table 3:** Respective proportion of patients classified as microbiologically documented and clinical tuberculosis according to disease localization and HIV-status.

	Pulmonary TB				Extrapulmonary TB			
	HIV-infected	HIV-uninfected	HIV-unknown	Total	HIV-infected	HIV-uninfected	HIV-unknown	Total
	(n = 93)(n = 270)(n = 293)(n = 656)				(n = 38)(n = 46)(n = 67)(n = 151)			
	Percentage (95% CI)				Percentage (95% CI)			
**Documented**	61.3 (50.6–71.2)	84.1 (79.2–88.2)	83.6 (78.8–87.7)	80.5 (77.3–83.5)	47.4 (31.0–64.2)	63.0 (47.6–76.8)	41.8 (29.9–54.5)	49.7 (41.1–57.9)
**Clinical**	**38.7** (a)(28.8–49.4)	15.9 (11.8–20.9)	16.4 (12.3–21.1)	19.5 (16.5–22.7)	52.6 (35.8–69.0)	**37.0** (b)(23.2–52.5)	58.2 (45.5–70.2)	**50.3** (a)(42.1–58.6)

TB: tuberculosis; HIV: Human Immunodeficiency Virus; CI: confidence interval.

(a) *p*<0.0001;

(b) *p* = 0.03; TB: tuberculosis.

### Microbiological Tests

#### Conventional assays (SM and solid-culture)

Most patients with suspected pulmonary TB had 3 sputum samples submitted for Smear Microscopy (SM) and culture. Extrapulmonary samples were harvested on at least one occasion (CSF, pericardial, pleural or ascitis fluid, urines). Respiratory specimens were collected in aseptic tubes and processed by the N-acetyl-L-cysteine (NALC)-NaOH method before SM and culture.

•SM: Smears were stained using the hot Ziehl-Neelsen (ZN) method according to standard guidelines and read using light microscopy. The semi-quantitative yield of AFB was recorded according to WHO recommendations [17]. Positive results were defined by an AFB number above the threshold for positivity (i.e., 10 AFB/100 HPF on the IUATLD/WHO scale); and negative results by a smear with no AFB/100 HPF (high power field). Moreover, among positive scores, scanty results were included and defined as 1–9 AFB per 100 HPF. The SM results were analyzed by sputum samples at their highest level [18], [19]. Diagnostic smears were those performed at enrollment and follow-up smears were those performed during treatment.
•Mycobacteria growth detection:
  1. Two culture techniques were used:
    - *∘ Solid media*: 0.2 mL of each decontaminated and reconstituted sample were inoculated in one tube of Lowenstein-Jensen (LJ) (bioMérieux India Inc.) and incubated at 37°C. Tubes were periodically observed for microbial growth. The enumeration of numbers of colonies per tube and time to detection (TTD) were recorded.
    - *∘ Automated liquid media*: 0.5 mL of reconstituted BacT/ALERT® MAS fluid was added to BacT/ALERT® MP bottle (bioMérieux India Inc.) and 0.5 mL of each decontaminated and reconstituted sample were inoculated to the prepared BacT/ALERT® MP bottle. The inoculated bottles were loaded in the BacT/ALERT® 3D or MB/BacT® system. Detection of mycobacterial growth was monitored automatically and the time of first positivity of the vials during the culture was recorded for each inoculated bottle. The Time to Detection (TTD) of mycobacterial cultures was defined as the day that the bottles gave a positive Growth Index (GI) reading and ZN staining demonstrated AFB bacilli.
  2. The AFB growth was confirmed by both the LJ medium and BacT/ALERT® MP flagged positive bottle using smear ZN staining.
  3. Identification of the bacilli as *M.tuberculosis* complex was performed using a rapid DNA hybridization test specific for *M.tuberculosis* complex (Accuprobe MTBC; GenProbe Inc., San Diego, CA, USA). Two ml were drawn from positive liquid media and centrifuged for 20 min at 3,500×*g,* and the pellet was used in the hybridization test. Samples were read in a Leader 50 luminometer (Gen-Probe Inc. San-Diego, CA, USA). Samples producing signals greater than 30,000 RLUs (Leader 50 luminometer) were considered positive. Cultures were probed on the first working day following the MB/BacT instrument positive flag. Starting from the evidence of a very high number of AFB in the MB/BacT medium from the first signal, this procedure was adopted to shorten the identification time. Extended species Identification of the MOTTs (mycobacteria other than *M.tuberculosis)* was not performed.
  4. All positive cultures were stored and kept at −20°C for future studies.

**Table 4:** Demographic and clinical characteristics of the non-active TB individuals.

	Individuals without active TB (n = 980)							
	Low risk of TB exposure		High risk of TB exposure					
	(n = 559)		(n = 421)					
	Blood donors	Healthy Community Adults	Non -TB Patients (a)	Health Care Workers	Healthy Family Contacts	Cured TB		
	(n = 459)	(n = 100)	(n = 73)	(n = 123)	(n = 135)	(n = 90)		
	**Percentage**							
**Pulmonary disease**	NA	NA	58.9	NA	NA	100.0		
**Median Age (IQR)**	NA	44.0	40.0	30.0	32.0	34.0		
**Ratio M/F**	NA	1.7	2.5	1.0	1.0	3.1		
**Male prevalence**	NA	63.0	71.2	49.6	51.1	75.6		
**Diabetes**	0.0	0.0	20.9	0.8	0.0	0.0		
**Poor Nutrition**	0.0	0.0	0.0	0.0	0.0	0.0		
**Past TB**	NA	0.0	10.9	0.0	0.7	100.0		
TB <2 years	NA	0.0	1.4	0.0	0.0	28.1		
TB 2 to10 years	NA	0.0	5.5	0.0	0.0	71.9		
TB>10 years	NA	0.0	4.1	0.0	0.7	0.0		
**Contact with TB**	0.0	0.0	4.7	0.0	100	0.0		

(a) The hospitalized non-active TB control group presented a pulmonary disease in 58.9% of the 73 enrolled individuals with a definitive diagnosis excluding active TB: bacterial or atypical pneumonia (34.9%); Bronchitis (11.6%), MOTT infection (11.6%), lung cancer (7.0%), COPD (7.0%), complicated Asthma (7.0%), Lung abscess (4.7%), post-TB Lung fibrosis (2. 3%), Mediastinal lymphadenopathy (2.3%) and Sarcoïdosis (2.3%);

### New Tests

- Blood culture: around 3–5 mL of blood from HIV-positive patients with suspected TB were either collected in a sterile SPS or heparinized tube, or directly inoculated into a BacT/ALERT® MB blood culture bottle at the patient’s bedside. The inoculated bottles were immediately transferred to the microbiological laboratory for loading in the BacT/ALERT® 3D or MB/BacT® system for incubating and monitoring mycobacterial growth. Identical procedures for the TTD and mycobacterial identification were performed as above.
- NAAT assays: Amplified *Mycobacterium tuberculosis* Direct (AMTD) test (GenProbe Inc., San Diego, CA, USA): GenProbe MTD analysis was performed from the processed specimen in case of sputum and other non- sterile specimens using the required volume as indicated by the manufacturer.

### Immunological Tests

HIV-infection was diagnosed by two ELISA (Retroquic Comb Aids-RS, Span Diagnostics, India and HIV TRI-DOT, J. Mitra & Co, India) in serum. The person was considered HIV-positive when the serum was reactive in both tests if a serum was reactive in only one ELISA, HIV-Western Blot was done as a confirmatory test to rule out a false ELISA test result [3].

### Data Collection

These data were subsequently transferred to EPIINFO files by one of the authors (ST). Each file included the patient’s characteristics (serial number, study center, date of enrollment, nature of specimen collected, patient study group, age, sex, permanent address), risk of TB, clinical symptoms (cough for more than 2 weeks, persistent low-grade fever –higher than 37.5C for 2 weeks-, weight loss -more than 10% of the usual weight within the last 3 months-, night sweats, anorexia, fatigue, dyspnea, chest pain and haemoptysis), whether TST was performed or not (if performed, the diameter of induration was recorded), chest X-ray findings, the effect of a 10-day antibiotic trial, the final clinical diagnosis, the CSTB, the final therapeutic intervention with the therapy initiation date and the anti-TB drugs prescribed. The last part of the file consisted of treatment outcome obtained during the 6 to 9 month follow-up of each individual with TB (clinical symptoms relief, chest X-rays and microbiological conversion) and the absence of clinical symptoms in the control groups. Patients with both pulmonary and extrapulmonary localizations (infiltrate and pleural effusion, for instance) were classified as pulmonary TB.

**Table 5:** Frequency of smear microscopy positive results of patients with active-TB according to their disease localization and HIV-status.

	Pulmonary TB		Extrapulmonary-TB		Pulmonary vs Extrapulmonary
	AFB-positive/total number of patients tested per group (Percentage)	Comparison by HIV Status *p* value	AFB-positive/total number of patients tested per group (Percentage)	Comparison by HIV Status *p* value	*p* value
**HIV-infected**	38/90* **(42.2%)**	0.0001 vs HIV-uninfected	3/31** **(9.7%)**	0.34 vs HIV-uninfected	0.0004
		0.0047 vs HIV-unknown		0.37 vs HIV-unknown	
**HIV-uninfected**	205/270 **(75.9%)**	0.0002 vs HIV- unknown	9/46 **(19.6%)**	1.00 vs HIV-uninfected	0.002
**HIV-unknown**	180/293 **(61.4%)**	–	13/67 **(19.4%)**	–	0.0001
**Total**	423/653* **(64.8%)**		25/144** **(17.4%)**		0.0001

3 patients had no AFB records;

** 7 patients had no AFB records.

**Table 6:** Frequency of smear microscopy positive results of adult high-risk controls studied.

AFB-positive/total number of subjects tested per group (Percentage)			
No TB patients	Health Care Workers	Healthy Family contacts	Cured TB patients
4/73 **(5.5%)** ^a)^	0/123 (0.0%)	0/135 (0.0%)	0/90 (0.0%)

TB: tuberculosis; HIV: Human Immunodeficiency Virus; a) For comparison of the “no-TB group" with the “Health care group", or with “healthy family contacts" or “cured-TB patients" the p value were at least = 0.03; AFB: acid fast bacilli.

Active TB was defined “microbiologically confirmed" after identification of *M.tuberculosis* in LJ and/or in BacT/ALERT MP culture, molecular tests or histology. Conversely, patients were classified as having “clinical TB" if the diagnosis was based on clinical and radiologic criteria (after excluding other diseases) including appropriate responses to anti-tuberculous therapy.

**Table 7:** Comparative proportions of the semi-quantitative smear microscopy results (AFB score) obtained in groups of patients with active pulmonary TB enrolled at the different centers independent of their HIV-status.

	Smear microscopy AFB score							
	Positive over total (%)							
Center	0	Scanty	(+)	(++)	(+++)	(++) and (+++)	*p* value*	Total AFB(+)
**HINDUJA**	51/162 (31.5)	12/162 (7.4)	33/162 (20.4)	23/162 (14.2)	43/162 (26.5)	66/162 (40.7)	1	111/162 (68.5)
**AIIMS**	50/90 (55.6)	6/90 (6.7)	15/90 (16.7)	8/90 (8.9)	11/90 (11.8)	19/90 (21.1)	**0.0003**	**40/90 (44.4)**
**SAFDARJUNG**	51/95 (53.7)	1/95 (1.1)	14/95 (14.7)	16/95 (16.8)	13/95 (13.7)	29/95 (30.5)	0.07	**44/95 (46.3)**
**JALMA**	31/89 (34.8)	6/89 (6.7)	13/89 (14.6)	10/89 (11.2)	29/89 (32.6)	39/89 (43.8)	0.6	58/89 (65.2)
**NIZAM**	7/49 (14.3)	7/49 (14.3)	8/49 (16.3)	9/49 (18.4)	18/49 (36.7)	27/49 (55.1)	**0.05**	42/49 (85.7)
**TRC**	40/168 (23.8)	13/168 (7.7)	29/168 (17.3)	38/168 (22.6)	48/168 (28.0)	86/168 (51.2)	**0.01**	128/168 (76.2)
**TOTAL**	230/653 (35.3)	45/653 (6.9)	112/653(17.2)	104/653 (15.9)	162/653 (24.8)	266/653 (40.7)		423/653 (64.8)

AFB: acid fast bacilli.

* *p* value of the cumulative group (++ and +++) *versus* the mean of total enrolled patients with the same AFB score.

### Statistical Analysis

Median and interquartile (IQR) ranges were calculated. For continuous variables the Mann-Whitney U test or Wilcoxon for pair-wise comparisons were used. For categorical variables Chi square was used. Analysis was carried out with SPSS v 14 for Windows (SPSS Italia SRL, Bologna, Italy) and EPIINFO (CDC, Atlanta, USA).

**Table 8:** Comparative proportions of the semi-quantitative smear microscopy results (AFB score) obtained in groups of patients with active pulmonary TB according to their HIV status.

	Smear microscopy AFB score							
	Positive over total (%)							
HIV status	0	Scanty	(+)	(++)	(+++)	(++) and (+++)	*p* value*	Total AFB(+)
**HIV-infected**	52/90 (57.8)	6/90 (6.7)	11/90 (12.2)	14/90 (15.6)	7/90 (7.8)	21/90 (23.3)	0.0017	38/90 (42.2)
**HIV-uninfected**	65/270 (24.1)	25/270 (9.3)	49/270 (18.1)	51/270 (18.9)	80/270 (29.6)	131/270 (48.5)	0.034	205/270 (75.9)
**HIV-unknown**	113/293 (38.6)	14/293 (4.8)	52/290 (17.7)	39/293 (13.3)	75/293 (25.6)	114/293 (38.9)	0.616	180/293 (61.4)
**TOTAL**	230/653 (35.2)	45/653 (6.9)	112/653 (17.2)	104/653(15.9)	162/653 (24.8)	266/653 (40.7)		423/653 (64.8)

AFB: acid fast bacilli; HIV: Human Immunodeficiency Virus;

* *p* value of the cumulative group (++ and +++) in HIV-infected *versus* the mean of total enrolled patients with same AFB score.

## Results

### Characteristics of the Enrolled Population

Two thousand two hundred and thirteen (2213) subjects were enrolled in the 2-year period. Seventy-five adults were not included because their files were incomplete (**Figure 1** **).** Additionally, 272 children were evaluated separately [20], while 79 adults were included in a satellite study [21]–[23]. The remaining files correspond to 807 patients with active TB: 131 HIV-infected, 316 HIV-uninfected and 360 with unknown HIV-status. Among the 980 adults without active TB (controls), we identified 6 groups according to their characteristics. The “non active TB group" was necessary to evaluate the specificity and the predictive values of the different tests used in the present manuscript and in those in preparation.

**Table 9:** Impact of the physicians’ clinical suspicion of TB (CSTB) on the sensitivity of smear microscopy in tuberculosis patients according to disease localization and HIV status.

	Pulmonary TB					Extrapulmonary TB				
	TB diagnosis*CSTB LowCSTB HighCSTB Very High				*p* value “TB diagnosis vs very high"	TB diagnosisCSTB LowCSTB HighCSTB Very High				*p* value “TB diagnosis vs very high"
	N of AFB-positive/N of patients within the group					N of AFB-positive/N of patients within the group				
			(%)				(%)			
**HIV-infected**	38/90	0/1	21/56	18/32	0.2	3/31	0/2	2/19	1/10	1
	(42.2)	(0)	(37.5)	(56.2)		(9.7%)	(0)	(10.5)	(10.0)	
**HIV-infected**	205/270	0/1	39/83	167/186	0.0002	9/46 (	-	7/37	2/9	1
	(75.9)	(0)	(47.0)	(89.8)		19.6%)		(18.9)	(22.2)	
**HIV-unknown**	180/293	0/5	48/134	133/154	0.0001	13/67	0/2	11/58	2/7	0.6
	(61.4)	(0)	(26.1)	(74.8)		(19.4%)	(0)	(19.0)	(28.6)	
**Total**	423/653	0/7	107/273	318/372	0.0001	25/144	0/4	20/114	5/26	0.7
	(64.8)	(0)	(39.2)	(85.5)		(16.6)	(0)	(17.5)	(19.2)	

TB: tuberculosis; AFB: acid fast bacilli. *TB diagnosis by SM within the group considered; CSTB: clinical suspicion of TB

The demographic characteristics of the enrolled population and their risk factors for TB are shown in **Table 1** **–** **4** **.** Among the enrolled 807 active-TB patients, 81% had pulmonary-TB and 19% extrapulmonary-TB. Clinical diagnosis was retained in 24%, mostly in extrapulmonary-TB (43%) patients and in those HIV-infected (43%). Extrapulmonary TB was significantly more frequent in the HIV-infected (29%) patients compared to HIV-uninfected (14.6%; p = 0.0008) and HIV-unknown patients (18.6%; p = 0.018), with no significant difference between the latter two groups (p = 0.18) (**Table 1**). The localization of extrapulmonary TB is reported in **Table 2** **.** The proportion of patients classified as having microbiologically documented TB and clinical TB according to disease localization and HIV status is shown in **Table 3** **.** The frequency of clinical TB was significantly higher in extrapulmonary-TB patients than pulmonary-TB patients (*p*<0.0001) and in HIV-infected compared to HIV-uninfected and HIV-unknown pulmonary TB patients (p<0.0001). The final diagnosis of the hospitalized non–active TB control group is reported in **Table 4** **.**

**Table 10:** Comparative frequency of positive results of liquid versus solid culture in pulmonary and extrapulmonary active tuberculosis patients according to HIV status.

	Pulmonary TB				Extrapulmonary-TB			
	Liquid culture	Solid culture	Total	*p value of* liquid vs solid cultures*	Liquid culture	Solid culture	Total	*p value of* liquid vs solid cultures*
	Positive over total (%)				Positive over total (%)			
**HIV-infected**	55/86**	38/87**	56/87**	0.3	14/30	1/33	15/33	0.2
	(64.0)	(43.7)	(64.4)		(46.7)	(30.3)	(45.5 )	
**HIV-uninfected**	218/269**	184/268**	225/270	0.0001	27/46	20/45	29/46	0.2
	(81.0)	(68.7)	(83.3)		(58.7)	(44.4)	(63.0)	
**HIV-unknown**	238/293	164/293	245/293	0.0001	39/67	27/67	39/67	0.03
	(81.2)	(56.0)	(83.6)		(58.2)	(40.3)	(48.2)	
**Total**	511/648	386/648	526/650	0.0001	80/143	57/145	83/146	0.004
	(78.9)	(59.6)	(80.9)		(55.9)	(39.3)	(56.8)	

TB: tuberculosis; HIV: Human Immunodeficiency Virus; CI: confidence interval. *:*p* value of Comparison. **missing recorded culture;

Regarding the symptoms, in pulmonary-TB the prevalence of persisting cough was significantly lower in HIV-infected (73.1%) compared to HIV-uninfected (90.3%) or HIV-unknown status (93.0%) patients (p = 0.0001). Regarding the radiological signs the prevalence of infiltrates was significantly lower in HIV-infected (17.7%) compared to HIV-uninfected (53.5%) and HIV-unknown pulmonary-TB patients (61.4%)(p<0.0001). In extrapulmonary-TB, pleural effusion was observed in half of the patients independent of the HIV-status. Finally, the rate of major clinical symptoms and radiological findings was not significantly different in confirmed TB compared to possible-clinical TB and was independent of their HIV-status.

**Table 11:** Comparative frequency of positive results of liquid versus solid culture in the adult high-risk controls studied.

	High risk controls			
	Liquid culture	Solid culture	Total	*p value of* liquid vs solid cultures
		Positive over total (%)		
**No TB patients**	7/73* (**9.6)**	4/73* (5.5)	8/73* (11.0)	**0.5**
**Health Care Workers**	0/2 (0)	0/2 (0)	0/2 (0)	NA
**Healthy Family Contacts**	1/3* (**33.3)**	0/3 (0)	1/3* (33.3)	NA
**Cured TB patients**	0/90 (0)	0/90 (0)	0/90 (0)	NA

NA: not available; TB: tuberculosis; HIV: Human Immunodeficiency Virus.

* Cultures were positive with Mycobacteria other than Tuberculosis (MOTT).

### Microbiological Results

All specimens were tested by SM and solid and liquid cultures; only a portion of them were evaluated by PCR (manuscript in preparation). Disseminated TB was evaluated using liquid culture from blood and performed only in HIV-infected patients.

**Table 12:** Influence of the physicians’ clinical suspicion score of tuberculosis on the frequency of *Mycobacterium tuberculosis* culture -positive results in active pulmonary and extrapulmonary TB patients according to their HIV status.

	Pulmonary TB					Extrapulmonary TB				
	TB diagnosis*	**CSTB Low**	**CSTB High**	**CSTB Very High**	***p*** ** value “TB diagnosis vs very high"**	TB diagnosis*	**CSTB Low**	**CSTB High**	**CSTB Very High**	***p*** ** value “TB diagnosis vs very high"**
	**(%)**					**(%)**				
**HIV-infected**	56/87*	0/1	36/55	20/31	1	15/33	0/2	7/21	8/10	0.07
	(64.4)	(0)	(65.5)	(64.5)		(45.5 )	(0)	(33.3)	(80.0)	
**HIV-uninfected**	225/270	0/1	41/83	164/186	0.1	29/46	NA	23/37	6/9	1
	(83.3)	(0)	(61.4)	(89.3)		(63.0)		(62.2)	(66.7)	
**HIV-unknown**	245/293	4/5	96/134	145/154	0.001	39/67	1/2	33/58	5/7	0.6
	(83.6)	(80.0)	(71.6)	(94.2)		(48.2)	(50)	(56.9)	(71.4)	
**Total**	526/650	4/7	183/272	339/371	0.0001	83/146	1/4	63/116	19/26	0.1
	(80.9)	(57.1)	(67.3)	(91.4)		(56.8)	(25.0)	(54.3)	(73.1)	

NA: not available; AFB: acid fast bacilli; TB: tuberculosis; HIV: Human Immunodeficiency Virus. *TB diagnosis by culture within the group considered. CSTB: clinical suspicion of TB.

#### Smear microscopy (SM)

All the enrolled individuals had an SM examination for at least one specimen (if positive): 49% had two specimens and 23% had three, however no records were found in the 3 with pulmonary TB or the 7 with extrapulmonary TB. Twenty four SM-positive patients were culture-negative (3.0%-24/790), with no significant difference between pulmonary (3.1%) and extrapulmonary TB patients (2.8%). About half of them were classified as scanty SM.

**Table 13:** Comparative frequency of Mycobacteria other than Tuberculosis Mycobacteria (MOTTs) isolated in culture-positive specimens of pulmonary and extrapulmonary TB patients according to their HIV status.

	Pulmonary TB		Extrapulmonary TB		Total	
	Positive MOTTs over total culture-positive (%)		Positive MOTTs over total culture-positive (%)		Positive MOTTs over total culture-positive (%)	
		*p* value for		*p* value for		*p* value for
**HIV-infected**	7/56 (12.5)	HIV-infected vs HIV-uninfected 0.06	4/15 (26.7)	HIV-infected vs HIV-uninfected 0.1	11/71 (15.5)	HIV-infected vs HIV-uninfected 0.007
**HIV-uninfected**	11/224* (4.9)	HIV-infected vs HIV-unknown 0.002	2/29 (6.9)	HIV-infected vs HIV-unknown 0.004	13/253 (5.1)	HIV-infected vs HIV-unknown 0.0001
**HIV-unknown**	5/245 (2.0)	HIV-uninfected vs HIV-unknown 0.1	0/39 (0)	HIV-uninfected vs HIV-unknown 0.1	5/284 (1.8)	HIV-uninfected vs HIV-unknown 0.05
**Total**	23/525 (5.4)		6/83 (7.2)	Total pulmonary vs extrapulmonary 0.2	29/608 (4.8)	

HIV: Human Immunodeficiency Virus; TB: tuberculosis; MOTTs: Mycobacteria other than Tuberculosis.

* missing recorded identification; TB: tuberculosis.

The overall sensitivity was significantly higher in pulmonary TB patients than in extrapulmonary TB patients (p<0.0001) (**Table 5**). The sensitivity varied according to their HIV-status, being significantly lower in HIV-infected than in HIV-uninfected and HIV-unknown patients. The sensitivity was significantly higher in HIV-uninfected compared to HIV-unknown pulmonary TB patients. Similarly, the sensitivity was lower in HIV-infected compared to HIV-uninfected and HIV-unknown extrapulmonary TB patients but the difference was not significant.

**Figure 2:**
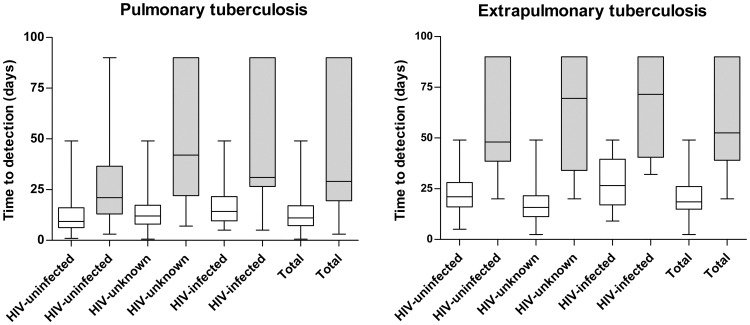
Comparative Time to Detection (TTD) of culture-positive specimens on solid (LJ) medium (grey bar) or with automated liquid (BacT/alert) (white bars) medium in active pulmonary and extrapulmonary TB patients according to their HIV status. TTD in days: median (IQR).

No specimens were obtained in the low-risk control groups. Specificity of SM was calculated for each high-risk control group: in the HCW, HFC and cured TB patients it was 100%, whereas in the hospitalized non-active TB patients it was 94.5% (**Table 6**). A significant difference in specificity was found between the hospitalized non-active TB patients and the other groups. In the hospitalized non-active TB patients, AFB-positivity, detected by culture, was associated with the presence of MOTT.

The Positive Predictive value (PPV) and the Negative Predictive value (NPV) of the SM were calculated with the specificity observed in non-active TB patients. In the pulmonary and extrapulmonary TB patients, the PPV was 0.991 and 0.862, the NPV was 0.233 and 0.367 and the likelihood ratio was 11.90 and 3.17, respectively.

**Figure 3:**
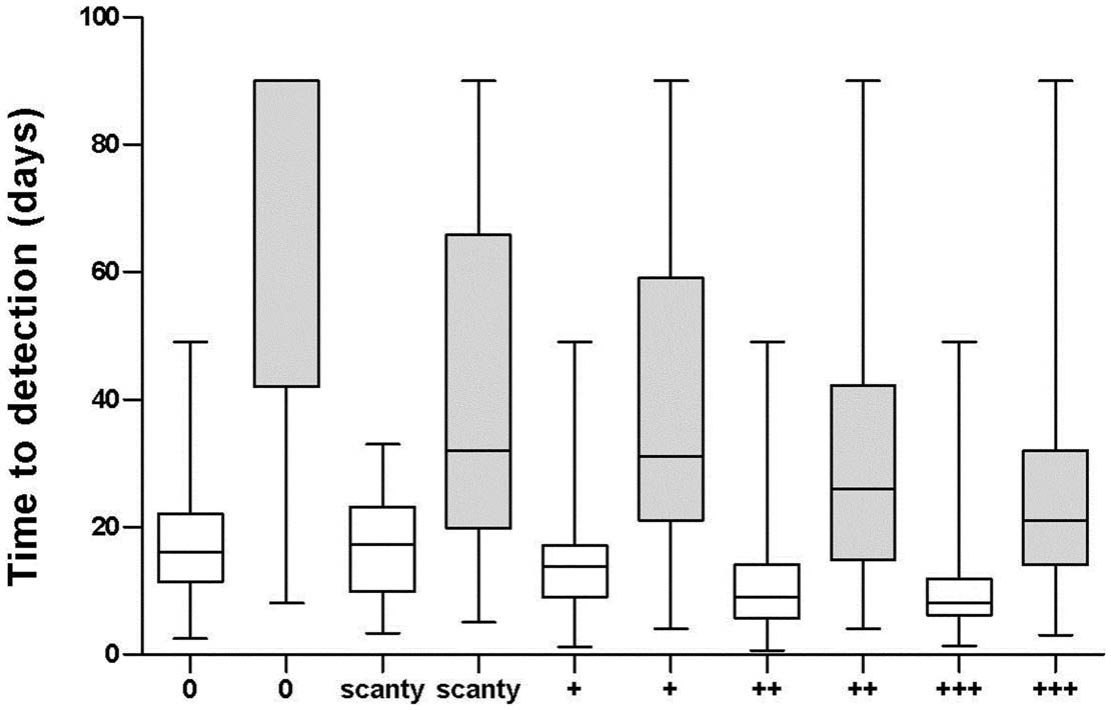
Time to Detection (TTD) of respiratory tract specimens cultured with the automated liquid culture BacT/ALERT MP (white bar) and with the Löwenstein-Jensen medium (grey bar) in relation to AFB scoring of TB patients. TTD in days: median (IQR).

SM sensitivity and the degree of infectivity may vary according to the populations of active TB patients enrolled at the different centers: the bacillary yield in sputum being directly related to the degree of infectivity and severity of the pulmonary disease [24]. AFB scores of the pulmonary TB patients enrolled at different centers are shown in **Table 7**. By combining the “++" and “+++" results, a gradient of infectivity was observed among the different centers: it was significantly higher in patients enrolled at Nizam’s and TRC, significantly lower at AIIMS and not different at Hinduja, Safdarjung and JALMA compared to the mean infectivity observed in the total enrolled population. AFB scores of the pulmonary TB patients varied also according to their HIV status as shown in **Table 8** **.** A significant lower infectivity was observed in HIV-infected than in HIV-uninfected TB patients (p<0.0001). No significant difference was observed between HIV-uninfected and HIV-unknown TB patients (p = 0.04).

The effect of the physician’s clinical suspicion of TB (CSTB) on SM sensitivity was evaluated according to TB localization and HIV status (**Table 9**). Compared to the global SM sensitivity observed in all pulmonary TB patients, a significantly higher sensitivity was observed in patients with a “very high" CSTB, (p<0.0001). However, the higher sensitivity observed in the HIV-infected patients with a “very high" CSTB was not significant (p = 0.2). No significant differences were found among those with extrapulmonary TB. The pre-test classification significantly increased the likelihood ratio in pulmonary TB patients (to 15.6; p<0.0001) but not in extrapulmonary TB patients (to 3.51, p = 0.8).

**Table 14:** Comparative contamination rate of liquid versus solid culture in pulmonary and extrapulmonary TB patients according to their HIV status.

Group	Pulmonary TB			Extrapulmonary TB			Total	
	Liquid culture	Solid culture		Liquid culture	Solid culture		Liquid culture	Solid culture
	Positive over total (%)		p value of liquid vs solid culture	Positive over total (%)		p value of liquid vs solid culture	Positive over total (%)	
**HIV-infected**	0/86 (0)	1/86 (1.2)	1	0/30 (0)	0/30 (0)	NA	0/116 (0)	1/116 (0.8)
**HIV-uninfected**	9/267 (3.4)	3/267 (1.1)	0.1	0/45 (0)	0/45 (0)	NA	9/312 (2.9)	3/312 (0.9)
**HIV-unknown**	5/293 (1.7)	7/293 (2.4)	0.7	1/67 (1.5)	0/67 (0)	NA	6/360 (1.6 )	7/360 (1.8)
**Total**	14/646 (2.2)	11/646 (1.7)	0.6	1/151 (0.7)	0/151 (0)	NA	15/797 (1.9)	11/797 (1.4)

HIV: Human Immunodeficiency Virus; TB: tuberculosis.

#### Culture of specimens

Since solid medium is recommended for culture in India, it was relevant to compare the performance of a solid and an automated liquid culture system using the same specimen from each enrolled patient. Their respective performances were evaluated using sensitivity, TTD and contamination rate.

### Diagnostic Values: Sensitivity, Specificity, NPV and PPV

The overall sensitivity of the liquid culture was significantly higher than the solid culture, both for pulmonary and extrapulmonary specimens, independent of the HIV status (**Table 10**). Among patients with pulmonary TB, the sensitivity of liquid and solid cultures was significantly lower in HIV-infected patients than the other groups (p<0.05); no significant difference was found in HIV-uninfected and HIV-unknown patients (p>0.05). Among patients with extrapulmonary TB, the sensitivity of the liquid and solid cultures was different only in HIV-unknown patients (p = 0.03). The cumulative sensitivity for the 2 culture methods was higher than that calculated for each methodology, although the difference was not significant (p = 0.7).

**Table 15:** Performances of the blood culture using the BacT/alert automated culture in the HIV-infected TB patients according to their TB localization.

Tuberculosis patients	Pulmonary TB	Extrapulmonary TB	Total
Positive over total (%)	7/55 (12.7)	4/32 (12.5)	11/87 (12.6)
**Median Time of positivity (IQR)(Days)**	23.0 (20.0–28.0)	27.0 (19.0–27.0)	23.0 (16.0–27.0)
**Median CD4+ cell counts/µL(IQR)**			
Negative culture	186 (95–232)	185 (83–250)	188 (79–223
Positive culture	121 (70–199)	154 (97–211)	121 (42–186)
Contamination rate (%)	9/55 (16.4)	7/32 (21.9)	16/87 (18.4)

HIV: Human Immunodeficiency Virus; TB: tuberculosis.

No specimens were harvested in the control low-risk groups (blood donors and HCA). The overall specificity (cumulative results of liquid and solid cultures) was calculated in each high-risk control group (**Table 11**). In the hospitalized non-TB patients, the specificity was 89% and was significantly lower than in cured TB patients (100%) (p = 0.01). Culture-positive results were associated with the isolation of MOTTs in the pulmonary specimens. The specificity in the other groups varied from 100% for the HCW group to 66.7% for the HFC group. The positive results were associated again with isolation of MOTTs in pulmonary specimens from the last group. All of these infected individuals were successfully treated, mostly with Clarithromycin.

The PPV and the NPV of the culture were calculated with the specificity observed in non-active TB patients. Respectively, in the pulmonary and extrapulmonary patients, the PPV was 0.985 and 0.922, the NPV was 0.344 and 0.512 and the likelihood ratio was 7.38 and 5.93.

The effect of the CSTB on the cumulative sensitivity of the culture (solid plus liquid) of the TB patients (**Table 12** **)** was further analyzed according to TB localization and their HIV status. Compared to the overall sensitivity of the culture observed in all pulmonary TB patients, a significantly higher sensitivity was observed in patients with a “very high" CSTB, (p<0.0001). However, the higher sensitivity observed in the HIV-uninfected patients with a “very high" CSTB was not significant (p = 0.1). No significant differences were found between HIV-infected patients and all subgroups of those with extrapulmonary TB. The pre-test classification increased the likelihood ratio in both pulmonary and extrapulmonary-TB patients (to 8.34 and 6.67, respectively) but the difference was only significant for pulmonary TB patients (p<0.0001).

### Species Identification

Identification of *M.tuberculosis* was performed using the Accuprobe assay in all culture-positive specimens. *M.tuberculosis* was found in almost all specimens (577/608, 94.9%) and MOTTs in 29 (4.8%) positive cultures (**Table 13**). The rate of MOTTs differed according to HIV status but not TB localization, which was significantly higher in the HIV-infected group compared to the HIV-uninfected and HIV-unknown groups. All the 29 active TB patients with MOTT isolation were successfully treated with anti-tuberculous therapy.

### Time to Detection (TTD)

The TTD was calculated for the positive culture in each group of active-TB patients according to their TB localization and HIV status. The median TTD (in days) with the solid culture (34.0; IQR: 21.0–0.012.0; IQR: 7.8–18.5) was significantly higher than that of the automated liquid culture (12.0; IQR: 7.8–18.5) and was independent of the TB localization and HIV status (p<0.0001) (**Figure 2**). The TTD of MOTTs was also significantly shorter with the liquid culture (median and IQR, 12.8 and 8–23 days) than with the solid culture (63.0 and 27–90 days) (p<0.0001).

It has been demonstrated that the TTD with the automated liquid culture is inversely proportional to the sputum bacterial yield [25]. We further evaluated the relationship between the AFB score and the TTD of the two culture techniques (**Figure 3**). By regression analysis a highly significant inverse relationship was found between the AFB score and the mean TTD of each culture system (BacT/ALERT MP: R^2^ = 0.924; LJ: R2 = 0.885) (p<0.0001) and a significant difference was observed for all the categories of the mycobacterial yield (p<0.0001) (**Figure 3**).

### Contamination Rate

The overall contamination rate in the TB culture was very low for both the liquid (1.9%) and the solid cultures (1.4%) and the difference between the two systems was not significant (p = 0.5). There was no statistical difference between the different groups (**Table 14**). No contamination was observed in the 421 specimens from controls. The 26 patients with contaminated culture were treated successfully with anti-TB drugs.

### Blood Culture

Among the HIV-infected TB patients, a high frequency of dissemination was reported, evidenced by multi-organ infection and the presence of *M.tuberculosis* in the peripheral blood [26]. In this study (**Table 15**) the overall sensitivity of the blood culture was low with no statistically significant difference between pulmonary TB and extrapulmonary TB (p = 0.7). The TTD of the blood culture was not significantly different in pulmonary-TB compared to extrapulmonary-TB. The median number of circulating CD4+ cells/µL was lower in patients with positive blood culture than in those with a negative blood culture, but the difference was not significant (p = 0.4). Among the 11 HIV-infected patients with positive blood cultures, 2 were initially classified as “disseminated TB", 2 as TB meningitis, 1 as pleural effusion, and the remaining 6 as pulmonary TB. Among the 11 HIV-infected patients being positive for *M.tuberculosis* in the blood culture, in 4 cases the positive blood culture was the only microbiological confirmatory test available. The 7 other patients also had culture-positive pulmonary or extrapulmonary specimens. A particular point to be noted was the significantly higher contamination rate of the blood culture compared to those obtained with the liquid culture on other specimens (p<0.0001).

## Discussion

We present the results of a prospective multicenter study that was designed to investigate the diagnostic values of microbiological tools for active TB in India, a highly endemic country.

Several microbiological tools were systematically applied to each patient with suspected active TB: 2 conventional tools (SM and solid culture) and 2 relatively new tools, the automated liquid culture system (BacT/ALERT MP) and molecular identification on positive culture. Two other new tools were applied on a portion of patients: direct nucleic acid amplification on specimens and a blood culture on HIV-infected patients.

Detection of AFB in specimens has a crucial clinical and epidemiological importance in the diagnostic algorithm for TB in both low-incidence countries [5] and high-burden countries with limited resources [7]. AFB detection in pulmonary secretions identifies patients with the greatest potential for *M.tuberculosis* transmission [27], [28]. Sputum SM offers the triple advantage of speed, simplicity and low cost. In many countries with a high TB burden, it is the most appropriate and most accessible diagnostic tool [29].

The sensitivity of SM in our study was within the expected range, detecting about 2/3 of the active pulmonary TB cases in HIV-uninfected and only half of the HIV-infected patients. This confirms data generated in high-burden countries with limited resources [1] and in India where the 0.630 million new cases notified in 2010 were SM-positive pulmonary TB [1], [2]. Our study showed slightly higher global results for pulmonary TB with 65.2% SM-positive compared to 51.0% reported in India for the year 2010 [1]. However, the sensitivity of SM varied according to HIV status, TB localization and the site of enrolled patients, which might depend upon the type of population recruited.

As shown here, less than 50% of HIV-infected pulmonary TB patients and around 10% of extrapulmonary TB patients were SM-positive, confirming the concern about its low sensitivity in HIV-infected TB individuals [30], [31]. The main issue with HIV-infected TB patients is the inability of the host response to control *M.tuberculosis* replication with low degree of lung lesions and a substantial diagnostic delay that increase of disease severity and a higher death rate [32]–[35]. Moreover, the magnitude of SM-negative TB in HIV-infected patients is underestimated: many of these patients die before TB is diagnosed, as shown by autopsy studies in Mumbai (India) where TB is the leading cause of death in these patients [36].

Paucibacillary TB is also an important diagnostic issue and we showed that SM detected only 17.3% of extrapulmonary-TB cases, and, it was even less efficient in the HIV-infected cases. This low detection rate might be associated with TB diagnosis delay, poorer treatment outcomes and greater mortality. In our study, the mortality rate was significantly higher (p = 0.02) in HIV-infected patients with extrapulmonary TB (13.2%) compared to those with pulmonary TB (2.2%). The CNS-TB in HIV-infected patients was frequently associated with death (80%).

In our study, the SM specificity was very high when evaluated in two high-risk populations: 100% in both the HCW and HFC groups. However, specificity was 94.5% when evaluated in the hospitalized non-active-TB patients; all SM-positive patients had MOTT infection. In India where TB is highly endemic, the frequency of MOTTs should not be ignored and we observed several mixed infections in HIV-infected and HIV-uninfected subjects, mostly in those with pulmonary TB. A recent study performed in India has shown such mixed infections (TB plus MOTTs) in TB patients in whom specimens were tested with PCR techniques using specific probes [37].

The pre-test probability using the CSTB score showed a significantly higher sensitivity of SM in groups of patients classified with the highest CSTB score (p = 0.001) with a significantly higher likelihood ratio only for pulmonary TB patients.

LJ medium has traditionally been the gold standard for TB diagnosis in India and in other resource-limited settings, although liquid culture is the gold standard of care in industrialized countries [38]. As demonstrated in our study, the BacT/ALERT MP liquid culture system was able to speed up the final diagnosis of both pulmonary and extrapulmonary TB, significantly reducing the TTD compared to the solid LJ culture (p<0.0001). Moreover, the sensitivity was found to be significantly higher using the BacT/ALERT MP system compared to the solid culture in pulmonary TB patients, independent of their HIV status (*p*<0.0001). These results confirm the data obtained in several published studies [39]–[42]. Our study results also showed that using both solid and liquid cultures resulted in incremented sensitivity detecting 83.3% in HIV-uninfected patients and 64.4% in HIV-infected pulmonary TB patients.

In our study, the specificity was 100% in cured TB patients, but was significantly lower (89%) in the hospitalized non-active TB patients (0.01); all patients had MOTT infection.

The pre-test probability using the CSTB score showed a significantly higher sensitivity of the culture in groups of patients classified with the highest CSTB score (p = 0.001) with a significantly higher likelihood ratio only for pulmonary TB patients.

The frequency of MOTTs in pulmonary and extrapulmonary TB specimens was higher in HIV-infected than in HIV-uninfected and HIV-unknown TB patients. MOTTs were detected earlier and more frequently using the liquid culture than the solid culture. The presence of MOTTs in these active TB patients might be considered as a bystander of mixed infection or bystander contaminants. Because all these patients were treated successfully with conventional anti-tuberculosis drug regimens for 6 months; the second hypothesis seems to be more likely. As discussed above, MOTTs were isolated in around 10% of hospitalized non-active TB patients. These patients were cured using second-line macrolides. Such a high prevalence of MOTTs in non-active-TB patients has not been previously reported in India and only few reports reported isolation of MOTTs in HIV-infected [43] and HIV-uninfected patients [44]. In our study, MOTTs was identified using the molecular technique (AccuProbe) and no strain speciation was performed. The use of a combination of genus-specific PCR primers might be useful [37].

Automated non-radiometric liquid culture systems are able to continuously monitor the positive results and data suggested that the TTD of *M.tuberculosis* using the MGIT960 system was a viable alternative to colony counting [25]. We established that both the BacT/ALERT MP system and the LJ medium showed an inverse relationship between TTD and semi- quantitative AFB score of sputum: an increase in TTD was correlated with a decreased yield (Figure 3). Thus, besides the already described advantages of the liquid culture system, the TTD might be considered as an additional internal quality control system for the smear microscopy tool.

The use of an automated blood culture system provided a novel opportunity to diagnose mycobacteremia in HIV-infected patients [45]. In a recent Indian prospective study on 52 HIV-infected patients with suspected active TB, mycobacteria were isolated in sputa or fecal samples from 20 patients and in 9 of these patients mycobacteria were also isolated from their blood specimens [46]: the calculated overall sensitivity in that study was 17.1%, not significantly different (p = 0.6) from our results (**Table 15**). *M. tuberculosis* bacteraemia was detected in HIV-infected patients when disseminated disease was not suspected, but we also showed that blood culture was not necessary for the final diagnosis of TB in 2/3 of the HIV-infected patients. Similar results were obtained in a South African study, where 22% of 71 HIV-infected TB patients were positive for *M.tuberculosis* on blood culture, but 75% of them had AFB+ pulmonary isolates [47].

One of the issues of the liquid culture system is a higher contamination rate compared to those observed with the solid medium [48]. Our study showed a very low overall contamination rate, both with the MB BacT/Alert (1.9%) and with the LJ medium (1.4%) for both the respiratory and extrapulmonary samples. On the other hand, it was almost 10 times higher with the blood cultures using the MB BacT/Alert. There was no clear explanation apart from possible technical failures during the process of inoculation of the vials.

In conclusion, conventional microbiological tools lead to results similar to those already described in India; special features being that the HIV-infected TB patients were less detected by SM and culture. Also, due to high endemicity of TB in India, the detection of MOTT is of high significance: mixed infections of both TB and MOTT found in this study were found in most of the HIV- infected and HIV- uninfected patients with pulmonary TB. This is also supported by some other recent Indian studies. New microbiological assays like the automated liquid culture system showed an increase of accuracy and speed of identification without increasing the contamination rate.

## References

[1] World Health Organization [WHO] (2011) WHO global tuberculosis control: WHO report 2010. Geneva: World Health Organization.

[2] Revised National TB Control Programme (2009). TB India 2009. RNTCP status report. New Delhi, India: Ministry of Health and Family Welfare.

[3] Dewan PK, Gupta D, Williams BG, Thakur R, Bachani D, et al (2010) National estimate of HIV seroprevalence among tuberculosis patients in India. Int J Tuberc Lung Dis 14: 247–249. 20074420

[4] Dinnes J, Deeks J, Kunst H, Gibson A, Cummins E, et al (2007) A systematic review of rapid diagnostic tests for the detection of tuberculosis infection. Health Technol Assess 11: 1–196. 10.3310/hta11030 17266837

[5] Migliori GB, Hopewell PC, Blasi F, Spanevello A, Raviglione MC (2006) Improving the TB case management: The International Standards for Tuberculosis Care. Eur Respir J 28: 687–690. 17012627 10.1183/09031936.06.00097506

[6] Steingart KR, Ramsay A, Pai M (2007) Optimizing sputum smear microscopy for the diagnosis of pulmonary-TB. Expert Rev Anti Infect Ther 5: 327–331. 17547496 10.1586/14787210.5.3.327

[7] Keeler E, Perkins MD, Small P, Hanson C, Reed S, et al (2006) Reducing the global burden of tuberculosis: the contribution of improved diagnostics. Nature 444 Suppl 1 49–57. 10.1038/nature05446 17159894

[8] Pai M (2004) The accuracy and reliability of nucleic acid amplification tests in the diagnosis of tuberculosis. Natl Med J India 17: 233–236. 15638300

[9] Pai M, O’Brien R (2006) Tuberculosis diagnostics trials: do they lack methodological rigor? Expert Rev Mol Diagn 6: 509–514. 16824024 10.1586/14737159.6.4.509

[10] Pai M, Kalantri S, Dheda K (2006) New tools and emerging technologies for the diagnosis of tuberculosis: part II. Active tuberculosis and drug resistance. Expert Rev Mol Diagn 6: 423–432. 16706744 10.1586/14737159.6.3.423

[11] Ling DI, Flores LL, Riley LW, Pai M (2008) Commercial Nucleic-Acid Amplification tests for diagnosis of pulmonary-TB in respiratory specimens: meta-analysis and meta-regression. PLoS One 3: e1536. 18253484 10.1371/journal.pone.0001536 PMC2212137

[12] Espy MJ, Uhl JR, Sloan LM, Buckwalter SP, Jones MF, et al (2006) Real-time PCR in clinical microbiology: applications for routine laboratory testing. Clin Microbiol Rev 19: 165–256. 16418529 10.1128/CMR.19.1.165-256.2006 PMC1360278

[13] Blakemore R, Story E, Helb D, Kop J, Banada JP, et al (2010) Evaluation of the analytical performance of the Xpert MTB/RIF assay. J Clin Microbiol 48: 2495–2501. 20504986 10.1128/JCM.00128-10 PMC2897495

[14] Marlowe EM, Novak-Weekley SMN, Cumpio J, Sharp SE, Momeny MA, et al (2011) Evaluation of the Cepheid Xpert MTB/RIF assay for direct detection of Mycobacterium tuberculosis complex in respiratory specimens. J. Clin. Microbiol 49: 1621–1623. 10.1128/JCM.02214-10 PMC3122817 21289151

[15] Boehme CC, Nabeta P, Hillemann D, Nicol MP, Shenai S, et al (2010) Rapid molecular detection of tuberculosis and rifampin resistance. N Engl J Med 363: 1005–1015. 20825313 10.1056/NEJMoa0907847 PMC2947799

[16] Catanzaro A, Perry S, Clarridge JE, Dunbar S, Goodnight-White S, et al (2000) The role of clinical suspicion in evaluating a new diagnostic test for active tuberculosis. JAMA 283: 639–645. 10665704 10.1001/jama.283.5.639

[17] Enarson DA, Rieder H L, Arnadottir T, Trébucq A (2000) Management of tuberculosis: a guide for low-income countries. 5th ed. Int. Union against Tuberculosis and Lung Disease, Paris.

[18] American Thoracic Society/Centers for Disease Control and Prevention (2000) Diagnostic standards and classification of tuberculosis in adults and children. Am J Respir Crit Care Med 161: 1376–1395. 10764337 10.1164/ajrccm.161.4.16141

[19] World Health Organization (1998) Tuberculosis handbook. WHO/ TB/98.253. Geneva, Switzerland.

[20] Dayal R, Verma V, Sharma B, Kumar G, Kumar N, et al (2012) Diagnostic value of Interferon-gamma release assays (QuantiFERON-TB Gold© In Tube) in childhood tuberculosis. Indian J Pediatr 79: 183–187. 21706246 10.1007/s12098-011-0469-y

[21] Goletti D, Raja A, Kabeer BSA, Rodrigues C, Sodha A, et al (2010) IFN-gamma, but not IP-10, MCP-2 or IL-2 response to RD1 selected peptides associates to active tuberculosis. J Infect 61: 133–143. 20470822 10.1016/j.jinf.2010.05.002

[22] Goletti D, Raja A, Kabeer BSA, Rodrigues C, Sodha A, et al (2010) Is IP-10 an Accurate Marker for Detecting *M.tuberculosis*-specific Response in HIV-Infected Persons? PLoS ONE 5: 577. 10.1371/journal.pone.0012577 PMC2935361 20830287

[23] Kabeer BSA, Raja A, Raman B, Thangaraj S, Leportier M, et al (2011) IP-10 response to RD1 antigens might be a useful biomarker for monitoring tuberculosis therapy. BMC Infectious Diseases 11: 135. 21595874 10.1186/1471-2334-11-135 PMC3120672

[24] Shaw J B, Wynn-Williams N (1954) Infectivity of pulmonary-TB in relation to sputum status. Am Rev Tuberc 69: 724–732. 13148535 10.1164/art.1954.69.5.724

[25] Pfeiffer C, Carroll NM, Beyers N, Donald P, Duncan K, et al (2008) Time to detection of *Mycobacterium tuberculosis* in BACTEC systems as viable alternative to colony counting. Int J Tuberc Ling Dis 12: 792–798. 18544206

[26] von Gottberg A, Sacks L, Machala S, Blumberg L (2001) Utility of blood cultures and incidence of mycobacteremia in patients with suspected tuberculosis in a South African infectious disease referral hospital. Int J Tuberc Lung Dis 5: 80–86. 11263521

[27] Grzybowski S, Barnett G D, Styblo K (1975) Contacts of cases of active pulmonary-TB. Tuberculosis Surveillance Research Unit. Report no. 3. Bull Int Union Tuberc 50: 90–106. 1218291

[28] Behr MA, Warren SA, Salamon H, Hopewell PC, Ponce de Leon A, et al (1999) Transmission of *Mycobacterium tuberculosis* from patient’s smear-negative for acid-fast bacilli. Lancet 353: 444–449. 9989714 10.1016/s0140-6736(98)03406-0

[29] World Health Organization (1998) Laboratory services in tuberculosis control. Part II. Microscopy. WHO/TB/98.258. Geneva, Switzerland.

[30] Barnes PF, Bloch AB, Davidson PT, Snider DE Jr (1991) Tuberculosis in patients with human immunodeficiency virus infection. N Engl J Med 324: 1644–1650. 2030721 10.1056/NEJM199106063242307

[31] Perkins MD, Cunningham J (2007) Facing the crisis: improving the diagnosis of tuberculosis in the HIV era. J Infect Dis 196: S15–27. 17624822 10.1086/518656

[32] Bruchfeld J, Aderaye G, Palme IB, Bjorvatn B, Britton S, et al (2002) Evaluation of outpatients with suspected pulmonary-TB in a high HIV prevalence setting in Ethiopia: clinical, diagnostic and epidemiological characteristics. Scand J Infect Dis 2002 34: 331–337. 10.1080/00365540110080025 12069014

[33] Perkins M D, Small P M (2006) Admitting defeat. Int J Tuberc Lung Dis 10: 1. 16466029

[34] Archibald L K, den Dulk M O, Pallangyo K J, Reller L B (1998) Fatal *Mycobacterium tuberculosis* bloodstream infections in febrile hospitalized adults in Dar es Salaam, Tanzania. Clin Infect Dis 26: 290–296. 9502444 10.1086/516297

[35] Githui W, Nunn P, Juma E, Karimi F, Brindle R, et al (1992) Cohort study of HIV-positive and HIV-negative tuberculosis, Nairobi, Kenya: comparison of bacteriological results. Tubercle Lung Dis 73: 203–209. 10.1016/0962-8479(92)90087-Z 1477386

[36] Lanjewar DN, Duggal R (2001) Pulmonary pathology in patients with AIDS: an autopsy study from Mumbai. HIV Med 2: 226–71. 10.1046/j.1468-1293.2001.00079.x 11737408

[37] Gopinath K, Singh S (2009) Multiplex PCR assay for simultaneous detection and differentiation of *Mycobacterium tuberculosis*, *Mycobacterium avium* complexes and other Mycobacterial species directly from clinical specimens. J Appl Microbiol 107: 425–435. 19302308 10.1111/j.1365-2672.2009.04218.x

[38] Garcia FG, Angulo GP, Garcia FG, Quero JH, Maroto Vela MC, et al (2008) Evaluation of the MB/BACT automated mycobacteria culture system versus culture on Lowenstein medium. Clin Microbiol Infect 4: 339–343.

[39] Morgan MA, Horstmeier CD, De Young DR, Robers GD (1983) Comparison of a radiometric method (BACTEC) and conventional culture media for recovery of mycobacteria from smear-negative specimens. J Clin Microbiol 18: 384–388. 6194175 10.1128/jcm.18.2.384-388.1983 PMC270810

[40] Alcaide F, Benítez MA, Escribà JM, Martín R (2000) Evaluation of the BACTEC MGIT 960 and the MB/BacT Systems for Recovery of Mycobacteria from Clinical Specimens and for Species Identification by DNA AccuProbe. J Clin Microbiol 38: 398–401. 10618124 10.1128/jcm.38.1.398-401.2000 PMC88732

[41] Lee JJ, Suo J, Lin CB, Wang JD, Lin TY, et al (2003) Comparative evaluation of the BACTEC MGIT 960 system with solid medium for isolation of mycobacteria. Int J Tuberc Lung Dis 7: 569–574. 12797700

[42] Adler H, Straub C, Frei R (2005) Comparison of BacT/ALERT 3D, Lowenstein-Jensen medium and Middlebrook 7H10/7H11 biplate for recovering mycobacteria from clinical specimens. Eur J Clin Microbiol Infect Dis 24: 499–500. 15986232 10.1007/s10096-005-1362-2

[43] Oberoi A, Kaur H (2004) Comparison of rapid colorimetric method with conventional method in the isolation of mycobacterium tuberculosis. Indian J Med Microbiol 22: 44–46. 17642685

[44] Narang R, Narang P, Mendiratta DK (2009) Isolation and identification of nontuberculous mycobacteria from water and soil in central India. Indian J Med Microbiol 27: 247–250. 19584507 10.4103/0255-0857.53208

[45] Hanscheid T, Monteiro C, Melo Christo J, Marques Lito L, Salgado MJ (2005) Growth of *Mycobacterium tuberculosis* in conventional BacT/ALERT FA blood culture bottles allows reliable diagnosis of mycobacteremia. J Clin Microbiol 43: 890–891. 15695697 10.1128/JCM.43.2.890-891.2005 PMC548049

[46] Gopinath K, Kumar S, Singh S (2008) Prevalence of mycobacteremia in Indian HIV-infected patients detected by the MB/BacT automated culture system. Eur J Clin Microbiol Infect Dis 27: 423–431. 18189149 10.1007/s10096-007-0450-x

[47] von Gottberg A, Sacks L, Machala S, Blumberg L (2001) Utility of blood cultures and incidence of mycobacteremia in patients with suspected tuberculosis in a South African infectious disease referral hospital. Int J Tuberc Lung Dis 5: 80–86. 11263521

[48] Cornfield DB, Beavis KG, Greene JA, Bojak M, Bondi J (1997) Mycobacterial growth and bacterial contamination in the Mycobacteria Growth Indicator tube and BACTEC 460 culture systems. J Clin Microbiol 35: 2068–2071. 9230383 10.1128/jcm.35.8.2068-2071.1997 PMC229904

